# A neuroplastic deafferentation hypothesis for bipolar disorder

**DOI:** 10.1016/j.mehy.2015.09.023

**Published:** 2015-12

**Authors:** Jonathan Rogers, Jamie Mirams, Rashmi Patel

**Affiliations:** Medical Academic Unit, Broomfield Hospital, Court Road, Chelmsford, Essex CM1 7ET, United Kingdom

## Abstract

Bipolar disorder, characterised by extreme cyclical variations in mood between depression and mania, is a common, debilitating and sometimes fatal psychiatric condition with an unclear aetiology. In this paper we propose a hypothesis for the development of bipolar disorder through which neuroplastic changes in response to an index depressive episode leads to the amplification of subthreshold pleasurable stimuli that then drive conversion into a manic state. This ‘pleasure deafferentation hypothesis’ is reached through a discussion of the neuroscientific basis of deafferentation at the level of the neuron and its role in the development of various neurological and psychiatric phenomena before a case for deafferentation as applied to bipolar disorder is justified and its implications discussed.

## Introduction

**Fig. 1:**
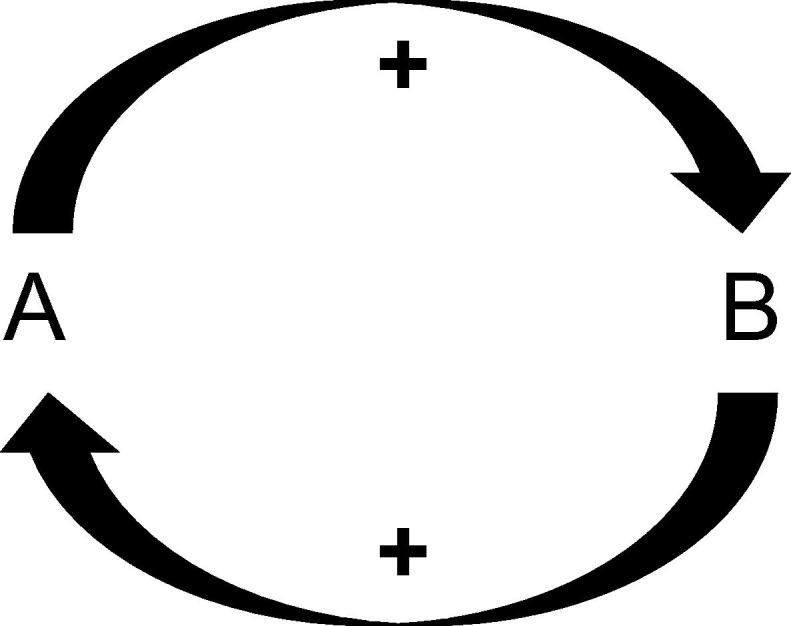
A positive feedback loop, in which a process A promotes a process B, which in turn induces A, such as the clotting cascade.

Many current theories in psychiatry are reliant on a notion of positive feedback, that is, a process ‘A’ acts to promote a second process ‘B’, which in turn augments process ‘A’ (Fig. 1). This is the basis of any theory that posits a vicious cycle or a mechanism that though initially exogenously stimulated subsequently becomes endogenously driven. At a neuronal level, this model is analogous to the ‘wind-up phenomenon’ [1], in which a neuron is sensitised by repeated stimulation, such that response to a given stimulus is actually enhanced; this occurs in nociception and thus is not an unreasonable place to start when considering less prosaic forms of ‘pain’. An example of this form of reasoning is present in the Beck depression hypothesis [2].

**Fig. 2:**
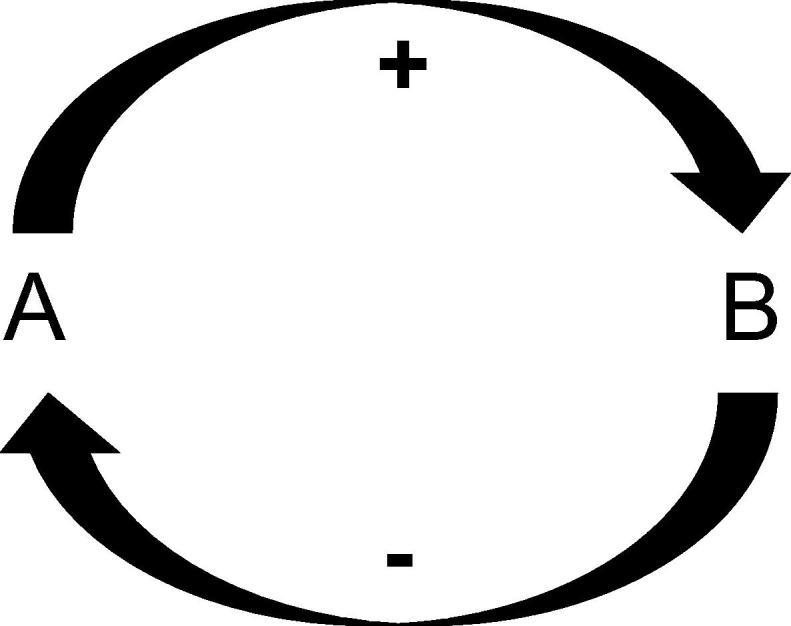
A negative feedback loop, in which A promotes B, which inhibits A, such as in the control of blood glucose levels with insulin.

However, whilst positive feedback is present and important at a neuronal level, it is generally less pivotal than and subservient to negative feedback systems (Fig. 2). Negative feedback underpins homeostasis and is crucial to any form of adaptation [3]. Perhaps in our theories of mental illnesses, we have somewhat underplayed this more fundamental mechanism.

The aim of this article is to examine the role of a specific form of negative feedback known as the deafferentation hypothesis, which states that when there is a substantial reduction in input to a neuronal pathway, this pathway adapts by responding to spurious signals as if they were true inputs. We shall first explain the neuroscience behind this rather counter-intuitive paradigm, before proceeding with a proof of concept using various examples from the fields of neurology and psychiatry. Then we shall examine the concept’s recent application to schizophrenia and finally explore how the deafferentation hypothesis applies to bipolar disorder.

## The hypothesis

After an extended period of depressive episodes, the brain becomes hypersensitised to pleasure and responds excessively and inappropriately to subthreshold signals by producing mania or hypomania. We propose that this mechanism of ‘pleasure deafferentation’ is responsible for the initiation of bipolar disorder.

## Background to the deafferentation hypothesis and proofs of concept

### The neuroscientific basis of the deafferentation hypothesis

It has long been appreciated that the developing nervous system is plastic; that is, that its architecture can be remodelled by a variety of endogenous and exogenous stimuli [1]. However, what is more recent is the idea of plasticity of the mature nervous system [1]. For instance, the auditory and somatosensory cortices are able to reorganise to inhabit locations that have previously been employed by cortex mapping other functions. Such mechanisms are said to be responsible for a huge range of symptomatology including allodynia, hyperalgesia, hyperpathia, vertigo, phonophobia and synkinesis [1].

The particular example of neuroplasticity that is relevant for our purposes is that which occurs when a neuron loses input. In this scenario, there are two possible outcomes: first, if all the input to a neuron is lost, it is likely to die; secondly, if there is some remaining input, the neuron is more likely to survive. However, in this second example, the surviving neuron will tend to be altered, sprouting axons around it and disinhibiting existing ineffective inputs [4].

At the subcellular level, there are several effects. The presynaptic terminal increases in size, with more vesicles, more of which are docked, a larger release zone, a larger readily releasable pool of neurotransmitters and an increase in the probability of release. At the postsynaptic terminal, there are more membrane receptors for the synaptic neurotransmitters and the membrane gives a greater response to a given applied current. In terms of individual neurotransmitters, there is an increase in NMDA (N-methyl-d-aspartate) response and a reduction in GABA-A (gamma-aminobutyric acid) and GABA-B response [4].

### Phantom limb pain

Following amputation or traumatic loss of a limb, it is not uncommon to develop pain referred to the absent limb. One study showed 69.7% of child and adolescent amputees had phantom sensations [5]. The pain in phantom limb syndrome is poorly responsive to conventional analgesia and is instead treated with antineuropathic agents. The same syndrome can result if merely the nerve supply to a limb is transected; in this case neuromas start to form, which have spontaneous activity. Moreover, the relevant dorsal root ganglia also show an increase in spontaneous activity. This is considered to be due to the upregulation of existing sodium channels and the formation of new channels. Tapping on the neuromas – that is, stimulating these sensitised pathways – is associated with increased activity in the afferent C-fibres and greater pain [6].

### Tinnitus

Tinnitus can also be viewed as a ‘phantom sensation’ [7]. Interestingly, both insufficient and excessive sound exposure can result in tinnitus [1]. The former case can easily be explained by a deafferentation hypothesis, in that an absence of sound results in fabrication of auditory meaning from irrelevant stimuli. This process seems to be time-dependent on the stimulus, in that temporary auditory deprivation leads to transitory tinnitus, but permanent deafness causes unremitting tinnitus [1]. The second case may either be due to the wind-up phenomenon, or may, perversely, also be due to deafferentation, since there is often subtle hearing loss present in tinnitus [8] and excessive sound exposure is known to cause partial deafness. Moreover, sufferers of tinnitus often report a distortion of actual sounds [1], possibly pointing to some more fundamental disruption of auditory processing.

Developing this hypothesis, De Ridder and colleagues found that 97% of patients with tinnitus did not experience the phenomenon in their dreams. They speculated that auditory processing involves generating a prediction of the environment before comparing it with external stimuli. Tinnitus, they propose, is due to deafferentation causing erroneous prediction. However, they suggest that during dreaming, this prediction error is not generated, abolishing the tinnitus [7].

### Charles-Bonnet syndrome

Charles-Bonnet syndrome is an ophthalmological condition characterised by visual hallucinations that occur in the context of visual impairment. It usually occurs in the presence of age-related macular degeneration, but has also been seen in glaucoma, cataract, optic neuritis and retinitis pigmentosa, among others. The hallucinations vary in complexity, but they tend to be time-limited, lasting for a few days [4].

Complete visual deprivation in those with previously normal sight is also known to produce hallucinations [9]. This has been induced experimentally with rapid results. In one demonstration, 13 normally sighted subjects wore opaque blind-folds for 5 days continuously. 10 of these participants experienced visual hallucinations, the majority starting within the first 24 h. These hallucinations ranged from simple spots of light to complex objects, landscapes and faces [10]. The proposed explanation is that an absence or reduction in normal visual input results in up-regulation of photoreceptors and downstream pathways, causing inappropriate neuronal activation that is interpreted as visual stimuli [4].

### Schizophrenia

Thus far, we have discussed deafferentation as a mechanism for abnormal sensory experiences, but it is quite a conceptual leap to discussing how persons with no sensory deficit develop multi-sensory psychotic experiences with marked emotional significance, as in schizophrenia.

In 2007, Ralph Hoffman, re-examined the established association between schizophrenia and social isolation. He was not new in noting the psychotogenic effects of social isolation: writing in 1959, Rosenzweig recounted stories from his experience in the US Air Force of young soldiers performing solitary guard for a night. The following morning, the young man would on several occasions be found believing he had heard voices, with confused thinking, perplexity and paranoia [11].

Hoffman’s explanation for this association was a social deafferentation hypothesis (SDA), positing that ‘high levels of social withdrawal/isolation in vulnerable individuals prompt social cognition programs to produce spurious social meaning in the form of complex, emotionally compelling hallucinations and delusions representing other persons or agents’ [12].

As well as finding social isolation itself to be associated with a higher risk of schizophrenia, there are also relationships with other factors such as immigration (especially in smaller groups), high social anhedonia scores and living conditions that foster isolation [12]. As further evidence for the role of social isolation, negative symptoms of schizophrenia often predate the positive symptoms and are also present as endophenotypes in the unaffected relatives of patients with schizophrenia [13]. This social isolation seems to be relevant later in the disease trajectory as well, since 80% of patients with auditory hallucinations claim that their hallucinations are worse when they are alone [14].

Neurobiological evidence for this process of deafferentation comes from the concept of synaptic ‘pruning’: there is a physiological process whereby around 30% of synaptic density is lost during adolescence in a process mediated by muscle-specific tyrosine kinase, agrin and neuregulin [15], but in schizophrenia this process seems to be excessive, resulting in auditory hallucinations [16]. It is possible that childhood stress, mediated by hypothalamo–pituitary adrenal axis hyperactivity, causes this synaptic elimination [17].

## The deafferentation hypothesis as applied to bipolar disorder

### The association between mania and depression

**Fig. 3:**
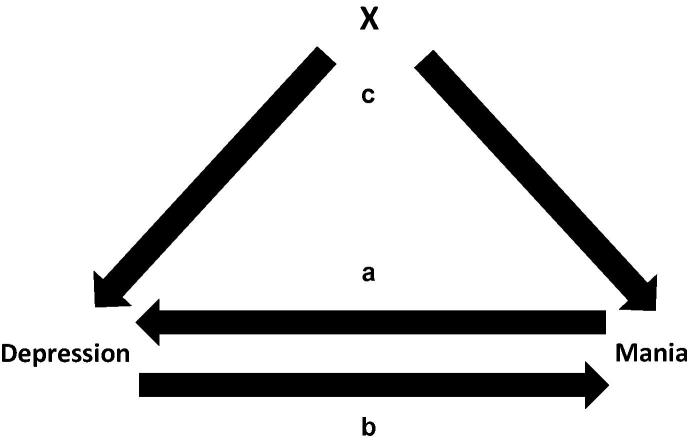
Putative causal relationships between mania and depression.

Mania is more common among those individuals who experience depression and depression is more common among those individuals who experience mania. There are three possible causal relationships that could link these two states, as illustrated in Fig. 3, which we shall examine in turn, along with the phenomenological predictions they would make.

In the first case (labelled a in Fig. 3), mania may cause depression. Bipolar disorder is often stated in these terms: “She became depressed because she regretted her actions,” or “He felt life held nothing for him after the mania”. This relationship would predict the following:

(1) Mania would predate depression;
(2) Mania would predominate over the course of the illness;
(3) The depressed phase is an artefact of the mania, rather than an entity in its own right, so would bear little relation to unipolar depression.

In the second case (labelled b in Fig. 3), depression may cause mania, giving the following predictions:

(1) Depression would predate mania;
(2) Depression would predominate over the course of the illness;
(3) The depression is the primary deficit and would closely resemble unipolar depression.

In the third case (labelled c in Fig. 3), depression and mania may both be caused by a third factor. This has an intuitive ring to it, as it seems apparent that at the core of bipolar disorder is some central dysregulation, permitting extremes of activity and mood. This theory would predict the following:

(1) Depressive and manic events would occur in proportion to each other;
(2) Depression and mania would co-dominate over the course of the illness;
(3) The depression is due to a core defect that is fundamentally different to that in unipolar depression, so would bear little relation to it.

### Evidence for a particular causal relationship between mania and depression

Let us now examine each of these predictions against the evidence we now have about bipolar disorder.

Firstly, depression nearly always predates mania in bipolar disorder. This tends to be by several years and is to such an extent that it is very common for patients who are diagnosed with bipolar disorder to have received a diagnosis of major depressive disorder previously [18], [19]. It does seem to be the case that there is a condition of unipolar mania, but it is less common than bipolar mania, comprising just 15.7% of people with mania in one study [20], and some cases are preceded by dysthymia [21], which would be consistent with our hypothesis. The course also tends to differ from bipolar disorder in terms of prognosis, depressive family history, age of onset and comorbidity [22]; thus unipolar mania may represent a distinct phenotype with a different pathological basis.

Secondly, whilst patients with bipolar disorder are euthymic (of normal mood) for about half of their illness, 80% of the remainder is depressive, whilst only 20% is manic or hypomanic (a less severe state than mania).

Thirdly, the depression seen in bipolar disorder is linked to unipolar depression in several respects. It is similar in symptomatology, bipolar depression being particularly similar to atypical depression [23]. It is related in heritability, with relatives of patients with both bipolar disorder and unipolar depression having higher rates of Major Depressive Disorder than the general population [24]. Finally, there are marked similarities in terms of neurobiology, brain-derived neurotrophic factor (BDNF) levels being reduced in both states [25]; in this study BDNF levels were further reduced in the people with bipolar disorder compared to those with unipolar depression. This echoes the familial findings that suggest a higher genetic loading in bipolar disorder [26].

### A pleasure deafferentation hypothesis for bipolar disorder

The question then is what the mechanism is whereby depression causes mania. We posit that this may be explained by the deafferentation hypothesis:

After an extended period of depressive episodes, the brain becomes hypersensitised to pleasure and responds excessively and inappropriately to subthreshold signals by producing mania or hypomania. At a neuronal level, this may correspond to action potentials firing in response to subthreshold dopaminergic inputs.

## Discussion

As in other deafferentation phenomena, the state is temporary, in this case possibly due to a re-accumulation of genuine pleasure input. Why then do not all depressed individuals develop mania? Our first response to this is that perhaps they do, but studies have not followed patients up for long enough. One landmark study lasting 27 years showed ‘conversion’ from unipolar depression to bipolar disorder to occur at a rate of about 1% per annum [27]. Most studies do not last this long and thus do not observe this effect.

Secondly, it is possible that some depressed individuals are more predisposed – genetically or otherwise – to this deafferentation effect than others. In particular, it seems that atypical depression (that is, depression with features of increased appetite, increased sleep and psychomotor retardation) is more associated with conversion from unipolar depression to bipolar disorder [28].

Thirdly, it is possible that a trigger is required to initiate the mania or hypomania. As we have noted on several occasions in this article, the process of deafferentation does not merely result in the fabrication of signal from noise, but it also distorts true inputs. In particular, small signals are augmented and amplified to have large effects. There is a wealth of literature on the triggers of mania and established precipitants include sleep deprivation [29], recreational use of stimulant drugs [30], antidepressants [31], [32], corticosteroids [31], anabolic steroids [32], falling in love [30] and childbirth [33]. In the presence of a de-afferented neuronal pathway, the effect of a pleasurable stimulus from falling in love or recreational stimulants could be greatly amplified. Childbirth may act in a similar way, as the rapid withdrawal of high levels of oestrogen leaves hypersensitised dopaminergic receptors [34]. Antidepressants may not always give pleasure *per se*, but they may act on precisely the monoamine pathways that we hypothesise become hypersensitised in depression. The role of steroids is likely more complex, but could explain why childhood abuse is associated with both HPA axis hyper-responsiveness [35] and with a poorer prognosis for bipolar disorder [36]. Cannabis use is also associated with the subsequent development of manic symptoms [37], though whether this is due to structural changes induced by cannabis or a common diathesis for substance misuse and bipolar disorder is unclear [38].

We hypothesise that, due to the proposed hypersensitisation of neural pathways mediating pleasure in depression, biochemical and psychological stimuli result in an excessive and disproportionate response, inducing mania.

There are limitations to this theory, which will need to be considered further. The place of mixed states (in which features of both mania and depression are seen) is problematic, as is the fact that there are some patients with bipolar disorder who seem to have an index manic episode. It possible that in this latter group, the patients are *de* sensitised rather than hypersensitised to pleasure, causing a relapse into subsequent depression. This could, indeed, explain the subsequent trajectory of bipolar disorder as a relapsing cyclical disorder.

In terms of psychopharmacology, deafferentation theory has an interesting explanation of the function of different medications. It has been established by a recent meta-analysis that some medications, such as lithium and quetiapine – and, less conclusively, lamotrigine, olanzapine and risperidone – reduce rates of both depressive and manic relapses [39]. Whereas the traditional explanation for this finding would be that these drugs act as ‘mood stabilisers’, reducing a underlying diathesis for affective instability, our hypothesis would instead claim that merely by reducing depression or mania, the incidence of the other could be reduced. Deafferentation explains how dopaminergic blockade – traditionally regarded as a pleasure and reward pathway – is effective in reducing mania. In terms of future drug development, a deafferentation theory would suggest developing any drug that reduces depression or mania without causing the other, in the expectation that it would in fact reduce the opposite pole. It may also suggest a gentle and gradual correction of an underlying deficit, rather than a dramatic one, which may precipitate another episode by deafferentation.

It is likely that this theory is not exhaustive and will not be relevant to all cases of the disease. However, we feel it merits further investigation. One method of testing our hypothesis could be comparing rates of mania in people with depression with healthy volunteers after taking antidepressants. Based on our hypothesis one would predict a higher rate of mania to result in depressed patients given antidepressant medications which would act as a trigger for conversion to mania. One final exciting prediction of our hypothesis is that as the neuroplasticity underlying the conversion of depressive to manic states is an adaptive phenomenon involving no tissue damage it could potentially be reversed, providing a target for future disease modifying therapies.

## Conclusion

We have discussed the deafferentation hypothesis, whereby a reduction in neuronal input to a pathway results in hypersensitivity of that pathway, causing noise and low-level signals to be amplified, resulting in disproportionate and inappropriate responses. We have explored the neuroscientific basis of the deafferentation hypothesis at the level of single neurons through its applications within neurology and psychiatry including the social deafferentation hypothesis of schizophrenia and proposed our own pleasure deafferentation hypothesis for bipolar disorder, which we feel is the most accurate model for initiation of the condition based on the current evidence.

## Sources of support

RP is supported by a UK Medical Research Council (MRC) Clinical Research Training Fellowship (MR/K002813/1).

## Conflict of interest statement

The authors declare no conflicts of interest.
